# Associations Between Health Literacy and Diabetes Self-Care Management Among Adults with Type 2 Diabetes in Southern Riyadh: A Cross-Sectional Study

**DOI:** 10.3390/healthcare14162532

**Published:** 2026-08-13

**Authors:** Mohammed Almutairi, Abdulaziz M. Alodhailah, Waleed M. Alshehri, Bader M. Almutairy

**Affiliations:** 1 Department of Medical Surgical Nursing, College of Nursing, King Saud University, Riyadh 11451, Saudi Arabia; 2 Community and Psychiatric Mental Health Nursing, College of Nursing, King Saud University, Riyadh 11451, Saudi Arabia

**Keywords:** health literacy, diabetes self-care management, Saudi Arabia, Riyadh, Orem’s self-care theory, chronic disease, HLS-Q12, SDSCA

## Abstract

**Highlights:**

**What are the main findings?**

- More than half of adults with type 2 diabetes attending primary healthcare centers in southern Riyadh had inadequate or problematic health literacy, highlighting a substantial gap in patients’ capacity to understand and manage their condition.
- Higher health literacy was associated with better diabetes self-care management, with older age and lower income linked to poorer health literacy and self-care outcomes.

**What are the implications of the main findings?**

- Routine health literacy assessment should be integrated into diabetes management in primary healthcare to identify patients requiring additional education and support.
- Tailored, health literacy-responsive interventions delivered by nurses and primary healthcare teams may improve diabetes self-management and help reduce health disparities among vulnerable populations in Saudi Arabia.

**Abstract:**

**Background:** Health literacy is widely recognized as a fundamental determinant of chronic disease self-management. Despite the escalating burden of diabetes mellitus in Saudi Arabia, the relationship between health literacy and diabetes self-care management within specific urban–peripheral communities remains inadequately characterized. This study aimed to examine the relationship between health literacy and diabetes self-care management among adults with diabetes residing in southern Riyadh, and to examine associations with key sociodemographic variables, including age, income, gender, and educational attainment. **Methods:** A non-experimental, cross-sectional, correlational design was employed. A purposive sample of 92 adults with a confirmed diagnosis of diabetes was recruited from primary healthcare centers in southern Riyadh. Data were gathered using the Arabic-validated 12-item European Health Literacy Scale (HLS-Q12) and the 8-item Arabic Summary of Diabetes Self-Care Activities (SDSCA). Descriptive statistics and Spearman rank-order correlations were computed using IBM SPSS Statistics, Version 25. **Results:** A significant positive correlation was identified between health literacy and diabetes self-care management (rs = 0.64, 95% CI [0.50, 0.75], *p* < 0.01). Income level demonstrated significant positive associations with both health literacy (rs = 0.41, *p* < 0.01) and self-care practices (rs = 0.40, *p* < 0.01). Age exhibited significant negative correlations with health literacy (rs = −0.36, *p* < 0.01) and self-care management (rs = −0.49, *p* < 0.01). Educational attainment was significantly associated with both self-care management (rs = 0.29, *p* < 0.01) and health literacy (rs = 0.26, *p* < 0.05). Gender was not significantly associated with either outcome. **Conclusions:** Health literacy was significantly associated with diabetes self-care management in southern Riyadh and may represent a potentially modifiable target for nursing intervention. Findings point to the urgency of designing equity-sensitive, culturally responsive interventions targeting older adults and economically marginalized populations. These preliminary findings may carry implications for nursing practice, diabetes education, and health policy in Saudi Arabia, pending confirmation in larger, prospective samples.

## 1. Introduction

Diabetes mellitus (DM) represents one of the most consequential chronic health challenges of the twenty-first century, with its global prevalence projected to affect approximately 643 million individuals by 2030 [1]. Within the Arabian Gulf region, and Saudi Arabia in particular, the epidemiological trajectory of diabetes has followed an accelerated course [2,3]. This trend is driven by rapid urbanization, demographic transitions toward sedentary lifestyles, and wholesale changes in dietary patterns that have accompanied the Kingdom’s economic transformation [4,5,6]. Saudi Arabia currently reports one of the highest age-adjusted diabetes prevalence rates globally, estimated at approximately 18.0% among adults, a figure that has intensified pressures on an already strained healthcare infrastructure [7,8].

Against this backdrop, the capacity of individuals with diabetes to engage consistently and effectively in self-care behaviors has emerged as a determinant of paramount clinical significance [9,10,11]. Effective diabetes self-care management encompasses a constellation of daily behaviors including dietary adherence, physical activity, blood glucose monitoring, foot care, and medication adherence [12,13,14]. The complexity and interdependence of these behaviors make diabetes self-management among the most demanding regimens in chronic disease care, requiring ongoing patient education and cognitive resources [15].

Health literacy occupies a central position in contemporary frameworks for chronic disease management [16,17]. It is defined by the World Health Organization as the cognitive and social skills that determine the motivation and ability of individuals to gain access to, understand, and use information in ways that promote and maintain good health [18]. Globally, studies employing these multidimensional instruments have documented that between 29% and 62% of adults demonstrate limited or problematic health literacy [19,20,21].

Inadequate health literacy is associated with misinterpretation of medical instructions, suboptimal medication adherence, and elevated rates of preventable hospitalizations [22]. Among individuals with type 2 diabetes, limited health literacy has been linked to suboptimal glycemic control and higher rates of complications such as nephropathy and retinopathy [23]. Saudi Arabia presents a distinctive context for these dynamics. Studies among Saudi adults have reported variable prevalence rates of limited health literacy ranging from 29% to 52%, depending on the population and the setting [24]. Evidence suggests that health literacy in the region is patterned by sociodemographic characteristics, where older adults and those with lower educational credentials demonstrate the lowest literacy levels [25].

Notwithstanding the extensive international evidence, critical gaps persist in the literature pertaining to Arabic-speaking populations in primary healthcare settings [26,27]. Existing studies in the region have frequently sampled from hospital-based populations or failed to account for the heterogeneity within Saudi Arabia’s geographic and socioeconomic landscape [27]. Southern Riyadh is characterized by lower average income levels relative to other metropolitan districts and a higher concentration of older adults, rendering it a population of distinct policy relevance. Yet, no prior study has specifically examined the interplay between health literacy and diabetes self-care management within this subpopulation.

Theoretical grounding for the present study is provided by Orem’s Self-Care Deficit Nursing Theory (SCDNT). This theory posits that effective self-care is contingent upon individuals possessing sufficient self-care agency, which is the acquired ability to engage in deliberate actions directed at maintaining health [28]. Within this framework, health literacy may be conceptualized as a constitutive dimension of self-care agency. When self-care agency is insufficient relative to the therapeutic self-care demand, a self-care deficit emerges, necessitating nursing intervention.

The present study therefore aimed to investigate the relationship between health literacy and diabetes self-care management among adults with diabetes in southern Riyadh. Secondary objectives included examining the associations between key sociodemographic variables, namely income level, age, gender, and educational attainment, and both health literacy and self-care management outcomes. The findings are intended to inform targeted nursing interventions and patient education strategies directed at reducing the burden of inadequately managed diabetes in an underserved urban Saudi Arabian community.

Based on these aims, the following hypotheses were formulated and tested. Primary hypothesis (H1): health literacy is positively associated with diabetes self-care management. Secondary hypotheses (H2 to H5): income level (H2), age (H3), gender (H4), and educational attainment (H5) are each associated with health literacy and self-care management scores. Consistent with the cross-sectional, correlational design, these hypotheses were framed and tested as associations rather than as predictive or causal (determinant) relationships; the study was not designed, and is not intended, to establish prediction models or causal determinants of health literacy or self-care behavior.

## 2. Materials and Methods

### 2.1. Study Design and Setting

This study employed a non-experimental, cross-sectional, correlational quantitative design to investigate the relationship between health literacy and diabetes self-care management among adults in southern Riyadh. Cross-sectional designs are appropriate for examining associations between variables at a single point in time and are widely employed in health services research to characterize the prevalence of health-related behaviors and their determinants within specified populations [29]. This design assumes that the relationship between health literacy and self-care management can be measured objectively and examined using statistical methods. Because the design is cross-sectional and observational, the study aim is to characterize the association between health literacy and self-care management, and their associations with sociodemographic variables; the design does not support prediction modeling or causal (determinant) inference, and no such claims are made.

Data were collected from participants recruited across five primary healthcare centers (PHCs) in the southern district of Riyadh, Saudi Arabia, following ethical approval, which was granted on 2 July 2024. This geographic focus was selected based on the documented socioeconomic characteristics of southern Riyadh, specifically, its comparatively higher rates of low educational attainment, lower average household incomes, and greater proportions of older adults relative to other Riyadh metropolitan districts, and the corresponding absence of published research examining health literacy and diabetes self-care management in this subpopulation.

### 2.2. Participants/Sample

Purposive sampling was employed to recruit adults who met the following inclusion criteria: (a) confirmed diagnosis of type 2 diabetes mellitus documented in medical records; (b) age ≥ 18 years; (c) Arabic language proficiency; (d) attendance at a southern Riyadh PHC during the data collection period; and (e) capacity to provide informed consent. Individuals with documented psychiatric diagnoses or cognitive impairments precluding meaningful self-report were excluded.

An initial sample of 120 participants was approached, of whom 92 provided sufficiently complete data for inclusion after exclusion of surveys with more than 30% missing responses. All 28 excluded surveys were removed because missing responses exceeded 30% of the total questionnaire items; no participants declined to participate after initial approach. Excluded participants appeared broadly similar to the included sample in terms of age, gender, and education level based on descriptive review; formal statistical comparison (e.g., chi-square or t-test) was not conducted. Full sample characteristics are presented in Table 1.

### 2.3. Measures/Instruments

#### 2.3.1. Health Literacy (HLS-Q12)

Health literacy was measured using the 12-item European Health Literacy Survey Questionnaire (HLS-Q12), an abbreviated version of the HLS-EU-Q47, validated for use in Arabic-speaking populations by Awwad et al. [30]. This validation was conducted in a sample of chronically ill adults in Jordan; a Saudi-specific psychometric validation of the HLS-Q12 has not yet been published, and this cross-national Arabic use is noted as a limitation. The HLS-Q12 operationalizes health literacy across four cognitive domains (access, understand, appraise, and apply health information) and yields a total score ranging from 12 to 48, with higher scores indicating greater health literacy. The Arabic version demonstrated robust internal consistency (Cronbach’s α = 0.832) and acceptable construct validity in prior validation studies [30]. In the present study, internal consistency of the HLS-Q12 was Cronbach’s α = 0.850. Reliability was calculated for the total HLS-Q12 score only; domain-level (subscale) reliability was not computed, as domain-level scores were not analyzed separately in this study.

#### 2.3.2. Diabetes Self-Care Management (SDSCA)

Diabetes self-care management was measured using the 8-item Arabic Summary of Diabetes Self-Care Activities (SDSCA-Arabic), adapted from the original instrument developed by Toobert et al. [15] and translated and adapted for use in Arabic-speaking populations. Unlike the HLS-Q12 (validated in Jordan), the SDSCA-Arabic was translated and psychometrically validated directly in a Saudi sample recruited from primary healthcare centers [31]. The SDSCA assesses the frequency of diabetes self-care behaviors over the preceding seven days across domains including diet, exercise, blood glucose monitoring, and foot care. In the present study, internal consistency was α = 0.81. Test–retest reliability of the SDSCA-Arabic (r = 0.912, *p* < 0.001) was reported in the original validation study [31]. As with the HLS-Q12, reliability for the SDSCA-Arabic was calculated for the total score only; domain-level reliability was not computed.

#### 2.3.3. Sociodemographic Questionnaire

A demographic questionnaire collected information on age, gender, marital status, educational attainment, employment status, monthly income, and diabetes disease duration.

### 2.4. Data Collection Procedures

Recruitment materials, including a study flyer and access letter, were distributed by email to administrators and nursing staff at the five participating PHCs, who disseminated them to eligible patients; recruitment was also supported through social media channels. Participants who chose to take part accessed the HLS-Q12, SDSCA-Arabic, and demographic questionnaire independently via a SurveyMonkey™ link; a hard-copy version of the survey was also made available but was not used by any participant. No reading or completion assistance from research staff is documented in the study protocol. Ethical procedures, including informed consent, anonymity, and data confidentiality, are described in Section 2.6.

### 2.5. Statistical Analysis

**Figure 1:**
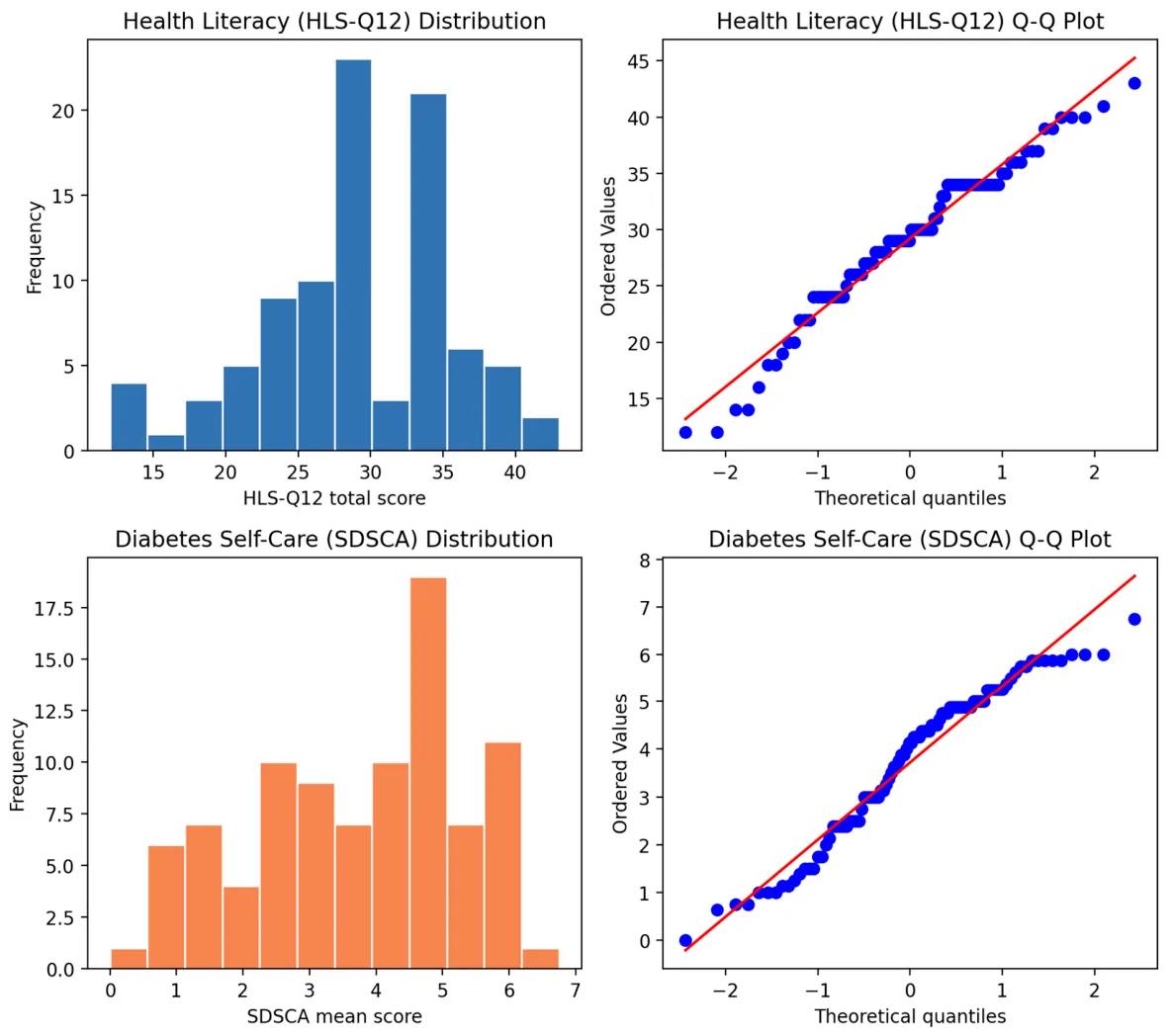
Distributional assessment of the two primary study variables (N = 92). (**Top row**): health literacy (HLS-Q12) histogram and Q-Q plot. (**Bottom row**): diabetes self-care management (SDSCA) histogram and Q-Q plot. Q-Q plots show ordered sample values against theoretical normal quantiles, with the red reference line indicating perfect normality.

Data were analyzed using IBM SPSS Statistics, Version 25 (IBM Corp., Armonk, NY, USA). Descriptive statistics, including frequencies, percentages, means, and standard deviations, were computed to characterize the sample. The one-sample Kolmogorov–Smirnov test was applied to assess distributional normality; results indicated that neither health literacy nor self-care management scores were normally distributed (*p* < 0.05), necessitating the use of non-parametric analytic approaches. Normality was further assessed using skewness, kurtosis, the Shapiro–Wilk test, and graphical inspection (histograms and Q-Q plots); full results of this expanded distributional assessment are reported in Section 3.2 and Figure 1. Spearman rank-order correlation coefficients (rs) were calculated to examine associations between health literacy, self-care management, and continuous sociodemographic variables. Income level and age were treated as continuous variables and education level as an ordinal variable, consistent with this rank-based method; gender was coded as a dichotomous variable (male/female), for which Spearman’s rho is mathematically equivalent to a point-biserial correlation. Effect sizes for all correlations were interpreted using Cohen’s conventional benchmarks (rs approximately 0.10 = small, 0.30 = medium, 0.50 = large). These benchmarks are a conventional heuristic rather than a universal or definitive standard, and effect-size interpretation should also consider the specific measures, population, and clinical context of this study. Internal consistency of both scales was confirmed using Cronbach’s α. Statistical significance was set at α = 0.05. An a priori power analysis was conducted using G*Power 3.1, based on a multiple linear regression model with five predictors (health literacy, gender, age, income level, and education level), a medium effect size (f^2^ = 0.15), a power of 0.80, and α = 0.05; this indicated a minimum required sample size of 92, which the achieved sample met. A post hoc sensitivity analysis using G*Power 3.1 (two-tailed Spearman correlation, α = 0.05, N = 92) further indicated that the primary association (rs = 0.64) was detected with >0.99 power. For the smaller secondary effects (rs = 0.26–0.29), achieved power ranged from 0.72 to 0.81, and these associations should be interpreted with corresponding caution.

### 2.6. Ethical Considerations

Ethical approval was obtained from the Institutional Review Board of King Saud Medical City (IRB registration number with KACST, KSA: H-01-R-053), Saudi Arabia, 2 July 2024. Participant anonymity was maintained through the use of SurveyMonkey™, a platform that does not collect identifying information such as IP addresses or email accounts. Written informed consent was obtained from all participants prior to data collection, and the voluntary nature of participation was emphasized. Data were stored on a password-protected server accessible only to the investigative team and will be destroyed after a five-year retention period.

## 3. Results

### 3.1. Sample Characteristics

The analytical sample comprised 92 participants, of whom 50 (54.35%) were female and 42 (45.65%) were male. The mean participant age was 42.09 years (SD = 14.61). The majority of participants were married (n = 68, 73.91%), and the largest educational subgroup had achieved secondary education (n = 32, 34.78%), followed by higher education (n = 30, 32.61%), primary education (n = 17, 18.48%), and intermediate education (n = 13, 14.13%). More than half of the participants were either not employed (n = 40, 43.48%) or retired (n = 18, 19.57%). The mean duration of diabetes diagnosis was 12.10 years (SD = 8.73), and the mean monthly income was 7270.23 Saudi Riyals (SD = 5621.90). The standard deviation of 562.19 reported in the original submission reflected a data-entry error and has been corrected here following verification against the raw dataset. Full sample characteristics are presented in Table 1.

### 3.2. Distributional Assessment and Health Literacy Classification

To supplement the Kolmogorov–Smirnov test reported in Section 2.5, distributional characteristics of the two primary study variables were assessed using skewness, kurtosis, the Shapiro–Wilk test, and graphical inspection (Figure 1). Health literacy (HLS-Q12) scores showed mild negative skew (skewness = −0.51) and approximately normal kurtosis (excess kurtosis = 0.13); the Shapiro–Wilk test indicated a significant departure from normality (W = 0.969, *p* = 0.025). Diabetes self-care management (SDSCA) scores showed a similar pattern of mild negative skew (skewness = −0.37) and platykurtic kurtosis (excess kurtosis = −0.92), with the Shapiro–Wilk test again indicating significant non-normality (W = 0.954, *p* = 0.003). The histograms and Q-Q plots in Figure 1 corroborate these results: both distributions show a broadly bell-shaped central tendency with departures from normality concentrated at the lower tail. Taken together, this converging evidence (formal test results, non-zero skewness and kurtosis, and graphical deviation from the normal reference line) supported the use of Spearman rank-order correlation rather than parametric alternatives. The bivariate relationship between health literacy and self-care management was additionally inspected for monotonicity; the scatterplot confirmed a positive, approximately monotonic association with no evidence of a non-monotonic or curvilinear pattern, no extreme or implausible values, and no missing data, supporting the appropriateness of a rank-based correlation approach for this dataset.

Based on established HLS-Q12 classification thresholds, 21 participants (22.8%) demonstrated inadequate health literacy (scores 12–27), 28 (30.4%) demonstrated problematic health literacy (scores 28–33), 27 (29.3%) demonstrated sufficient health literacy (scores 34–39), and 16 (17.4%) demonstrated excellent health literacy (scores 40–48). Accordingly, a combined 53.2% of participants exhibited inadequate or problematic health literacy, indicating a high prevalence of health literacy deficits within this sample. Table 2 summarizes the health literacy distribution.

### 3.3. Hypothesis Testing: Spearman Correlation Analyses

Hypothesis 1 proposed a significant positive relationship between health literacy and diabetes self-care management. The Spearman correlation analysis supported this hypothesis, revealing a statistically significant, large positive correlation (rs = 0.64, 95% CI [0.50, 0.75], *p* < 0.01), indicating that participants with higher health literacy scores consistently demonstrated greater engagement in diabetes self-care activities. Hypothesis 2 predicted significant positive associations between income level and both health literacy and self-care management. Results confirmed this hypothesis, with income demonstrating significant positive correlations with health literacy (rs = 0.41, 95% CI [0.22, 0.57], *p* < 0.01) and self-care practices (rs = 0.40, 95% CI [0.21, 0.56], *p* < 0.01). Hypothesis 3 proposed that older age would be associated with lower health literacy and poorer self-care management. This was confirmed: age exhibited significant negative correlations with health literacy (rs = −0.36, 95% CI [−0.53, −0.17], *p* < 0.01) and self-care management (rs = −0.49, 95% CI [−0.63, −0.31], *p* < 0.01). Hypothesis 4 predicted gender differences in health literacy and self-care management; however, gender was not significantly associated with either variable (health literacy: rs = −0.02, 95% CI [−0.22, 0.19], *p* = 0.873; self-care: rs = 0.11, 95% CI [−0.10, 0.31], *p* = 0.296); no significant association was observed for either variable. Hypothesis 5 proposed a significant association between educational attainment and both outcomes. Educational attainment was significantly associated with both diabetes self-care management (rs = 0.29, 95% CI [0.09, 0.47], *p* < 0.01) and health literacy (rs = 0.26, 95% CI [0.06, 0.44], *p* = 0.012); this pattern indicates full support for this hypothesis. Full results are presented in Table 3.

### 3.4. Multivariable Analysis of the Primary Association

To examine whether the association between health literacy and diabetes self-care management persisted after accounting for the sociodemographic variables most strongly linked to both outcomes, a parsimonious multiple linear regression was conducted with diabetes self-care management as the outcome and health literacy, age, monthly income, and education level entered simultaneously as predictors; gender was excluded given its non-significant bivariate association with both outcomes. Diagnostic checks supported the appropriateness of this model: variance inflation factors for all predictors were below 1.4, indicating no problematic multicollinearity; residuals were approximately normally distributed (Shapiro–Wilk *p* = 0.30) and homoscedastic (Breusch-Pagan *p* = 0.35). Because the outcome variable was not normally distributed, coefficient estimates were additionally verified using a bias-corrected bootstrap (5000 resamples); bootstrapped confidence intervals closely matched the asymptotic estimates reported below, supporting the robustness of the model to the non-normality of the outcome, Table 4.

Health literacy remained a significant, independent predictor of diabetes self-care management after adjustment for age, income, and education (B = 0.088, β = 0.355, *p* < 0.001): the primary association was not attributable to these sociodemographic factors. Age also remained an independent, significant predictor (B = −0.035, β = −0.311, *p* < 0.001). Income and education, by contrast, were no longer significant predictors once age and health literacy were accounted for (*p* = 0.063 and *p* = 0.088, respectively), despite each showing significant bivariate associations with self-care management. This attenuation is consistent with the moderate correlation observed between income and education (rs = 0.33, *p* < 0.01) and suggests that the bivariate associations of income and education with self-care management are partly explained by their shared variance with age and health literacy, rather than representing fully independent effects. The full model explained 45.0% of the variance in diabetes self-care management scores (adjusted R^2^ = 0.424).

## 4. Discussion

### 4.1. The Association Between Health Literacy and Diabetes Self-Care

The principal finding of this study, a significant positive correlation between health literacy and diabetes self-care management (rs = 0.64, 95% CI [0.50, 0.75], *p* < 0.01), aligns with the existing evidence base. Importantly, this association was not attributable to age, income, or education: the parsimonious multivariable regression reported in Section 3.4 confirmed that health literacy remained an independent, significant predictor of self-care management after adjustment for these sociodemographic factors (B = 0.088, β = 0.355, *p* < 0.001). Within Orem’s Self-Care Deficit Nursing Theory, this association is consistent with the proposition that health literacy forms part of the self-care agency an individual needs to meet the therapeutic self-care demand of diabetes; where that agency is limited, a self-care deficit, and correspondingly poorer self-care behavior, would be expected. This association, detected within a primary healthcare sample from an underserved urban–peripheral community in Saudi Arabia, reinforces the proposition that health literacy functions as a critical enabling resource for effective self-care engagement. Mechanistically, adequate health literacy may facilitate more accurate interpretation of blood glucose monitoring instructions, greater adherence to dietary and medication regimens, and more effective communication with healthcare providers, thereby translating into improved self-care practices [33]. The large effect size observed in this study is consistent with correlations reported by Suksatan et al. [34] in an Iranian primary healthcare context and by De Carvalho et al. [23] in a systematic review of health literacy in type 2 diabetes, providing cross-cultural validation for this association.

A distinctive finding of the present study concerns the high prevalence of inadequate or problematic health literacy within the sample, with more than half (53.2%) of participants demonstrating health literacy scores below the ‘sufficient’ threshold. This prevalence substantially exceeds estimates from comparable Saudi Arabian urban hospital-based samples, potentially reflecting the specific socioeconomic composition of southern Riyadh, where lower average educational attainment and income may compound health literacy deficits. This finding carries direct clinical implications, as it suggests that a substantial proportion of diabetes patients attending southern Riyadh PHCs may lack the informational competencies necessary to engage adequately in self-care. It further points to the imperative for healthcare providers, and nurses in particular, given their central role in patient education, to systematically screen for health literacy deficits and to deliver care using health literacy-universal approaches.

### 4.2. Sociodemographic Correlates of Health Literacy and Self-Care

The significant negative correlation between age and both health literacy (rs = −0.36, *p* < 0.01) and self-care management (rs = −0.49, *p* < 0.01) is a finding of considerable clinical and policy significance. Within the framework of Orem’s SCDNT, this pattern suggests that older adults in southern Riyadh may be particularly susceptible to self-care deficits arising from age-related reductions in health literacy-mediated self-care agency. This may reflect cohort effects in educational attainment, whereby older participants were less likely to have had access to formal education, as well as age-related cognitive changes that may reduce the ease with which complex health information is processed and applied. These findings align with those of Lee et al. [35], who documented declining health literacy among older adults with diabetes across multiple health systems, and carry urgent implications for the design of geriatric-sensitive diabetes education programs.

The significant positive associations between income level and both health literacy (rs = 0.41, *p* < 0.01) and self-care management (rs = 0.40, *p* < 0.01) are consistent with established social determinants of health frameworks, which position economic resources as enablers of health-promoting behaviors through multiple pathways including access to nutritious food, monitoring supplies, healthcare, and health information sources [36]. In the context of southern Riyadh, where income levels are comparatively lower than in other metropolitan areas, these findings suggest that economic vulnerability may amplify health literacy deficits and constrain self-care practices in ways that compound diabetes-related health inequities. The absence of a significant gender effect on health literacy and self-care management in this study diverges somewhat from prior Saudi Arabian studies that have reported gender differences [25], potentially reflecting the particular socioeconomic and demographic composition of the study population.

Educational attainment was significantly associated with both self-care management (rs = 0.29, 95% CI [0.09, 0.47], *p* < 0.01) and health literacy (rs = 0.26, 95% CI [0.06, 0.44], *p* < 0.05), consistent with the pattern observed for income and suggesting that socioeconomic resources broadly support both health literacy and self-care engagement. However, the multivariable analysis reported in Section 3.4 indicated that education’s association with self-care management was substantially attenuated once age, income, and health literacy were accounted for simultaneously (*p* = 0.088), suggesting that its bivariate association with self-care management is partly explained by overlap with these other socioeconomic and literacy-related factors rather than representing a fully independent effect. Formal education is often treated as a proxy for health literacy, but the two are conceptually distinct: health literacy also depends on prior experience with the healthcare system, the quality of health education received, and access to health information [33], and years of schooling alone may not fully capture these. Regardless of mechanism, the implication is that educational attainment alone is an insufficient surrogate for health literacy in clinical assessment, and that dedicated health literacy measurement is warranted in the care of individuals with diabetes.

### 4.3. Implications for Practice and Policy

These findings carry substantive implications for nursing practice, particularly in the domain of patient education and health promotion within primary care settings. Nurses are frequently the primary providers of diabetes education in PHC contexts and are uniquely positioned to deliver health literacy-responsive care. The Universal Precautions Approach to health literacy, which recommends that all clinical communication and educational materials be designed for maximum accessibility, regardless of assessed literacy level, represents a pragmatic and ethically sound framework for nursing practice in this context [37]. This includes using plain language and culturally resonant educational materials, incorporating teach-back techniques to confirm understanding, providing visual aids and pictorial guides, and ensuring that follow-up instructions are communicated verbally as well as in written form.

At the organizational and policy level, the findings support calls for the systematic integration of health literacy screening into diabetes care pathways in Saudi Arabian PHCs, using validated Arabic-language instruments such as the HLS-Q12. Routine health literacy assessment would enable the triaging of patients for targeted educational support and facilitate the tailoring of care plans to individual informational needs. Policymakers in the Saudi Ministry of Health may consider the development of national guidelines for health literacy-responsive diabetes care, alongside investment in community health education programs specifically targeting older adults, low-income households, and individuals with limited educational attainment in urban–peripheral communities such as southern Riyadh. These implications should be read as contextual, not substantive, advances: the study’s principal contribution is to document, for the first time, that the health literacy-self-care association previously reported elsewhere also holds in this specific, under-studied southern Riyadh population, rather than to establish a new mechanism or effect not previously described in the broader Saudi or Arabic-language literature. The geographic setting itself is therefore best understood as a contextual contribution. The multivariable analysis reported in Section 3.4, however, is a substantive methodological advance over the bivariate-only approach of the previous manuscript version: it demonstrates that the health literacy-self-care association is not attributable to age, income, or education, which bivariate correlation alone could not establish.

### 4.4. Limitations

Several methodological limitations of the present study merit acknowledgment. The cross-sectional design precludes causal inference; while a significant association between health literacy and self-care management was identified, the directionality and mechanisms of this relationship cannot be established from correlational data alone. The use of purposive convenience sampling from PHCs in southern Riyadh limits the generalizability of findings to the broader Saudi Arabian adult diabetes population, and selection bias cannot be excluded, as participants who attended PHCs may differ systematically from those who do not seek care. Self-reported data on self-care behaviors are susceptible to social desirability bias and retrospective recall limitations. Furthermore, the study did not assess several potentially confounding variables, including diabetes type, comorbidities, psychological well-being, social support, and access to diabetes education programs, all of which may independently influence the relationships examined. A further analytic limitation concerns the reliance on bivariate Spearman correlations for most reported associations. To address this for the study’s primary hypothesis, a parsimonious multivariable regression was conducted (Section 3.4), which confirmed that health literacy remained a significant, independent predictor of self-care management after adjustment for age, income, and education. However, the secondary sociodemographic associations reported in Table 3 (for example, between income and health literacy, or between education and self-care management considered on their own) remain unadjusted bivariate correlations; income and education were no longer independently significant once age and health literacy were accounted for in the multivariable model, indicating that some of these bivariate associations reflect shared variance among the sociodemographic variables rather than fully independent relationships. Readers should accordingly be cautious in attributing independent explanatory weight to any single sociodemographic variable based on Table 3 alone. Additionally, three further limitations merit acknowledgment. First, the reliance on PHC attendees introduces a systematic selection bias, as individuals who do not seek primary care may have lower health literacy and poorer self-care, suggesting that the prevalence of health literacy deficits reported here may underestimate the true population burden. Second, although purposive sampling was employed, the basis for participant selection beyond the stated eligibility criteria was not formally operationalised, which limits reproducibility. Third, chi-square goodness-of-fit tests examining the distributions of health literacy and self-care management categories were conducted as part of the broader doctoral research program from which this manuscript derives but are not reported here, as this manuscript focuses on the correlational analyses relevant to the study objectives.

## 5. Conclusions

This study provides the first systematic examination of the relationship between health literacy and diabetes self-care management among adults in southern Riyadh, Saudi Arabia, grounded within Orem’s Self-Care Deficit Nursing Theory. The identification of a significant, large positive correlation between health literacy and self-care management, alongside the high prevalence of inadequate health literacy within this community, highlights a critical and actionable gap in diabetes care. The age and income gradients identified here point toward older adults and economically marginalised households as the subpopulations requiring the most urgent attention. These findings contribute to the Arabic-speaking evidence base and have direct implications for Saudi PHC nursing practice and national diabetes education policy.

## Figures and Tables

**Table 1:** Sociodemographic and Clinical Characteristics of the Sample (N = 92).

Characteristic	Category	n (%)/Mean ± SD
Gender	Male	42 (45.65%)
	Female	50 (54.35%)
Age (years)		42.09 ± 14.61
Marital Status	Married	68 (73.91%)
	Single	18 (19.57%)
	Divorced	3 (3.26%)
	Widowed	3 (3.26%)
Education Level	Primary	17 (18.48%)
	Intermediate	13 (14.13%)
	Secondary	32 (34.78%)
	Higher Education	30 (32.61%)
Employment Status	Employed	34 (36.96%)
	Not Employed	40 (43.48%)
	Retired	18 (19.57%)
Duration of Diabetes (years)		12.10 ± 8.73
Monthly Income (SAR)		7270.23 ± 5621.90

Note: SAR = Saudi Arabian Riyals. Data are presented as frequencies and percentages for categorical variables and means ± standard deviations for continuous variables.

**Table 2:** Distribution of Health Literacy Categories Among Study Participants (N = 92).

Category	Score Range	n (%)
Inadequate	12–27	21 (22.8%)
Problematic	28–33	28 (30.4%)
Sufficient	34–39	27 (29.3%)
Excellent	40–48	16 (17.4%)
**Total**		**92 (100%)**

Note: Health literacy categories are based on HLS-Q12 scoring thresholds established by Sørensen et al. [32].

**Table 3:** Spearman Correlation Coefficients Between Sociodemographic Variables, Health Literacy, and Diabetes Self-Care Management (N = 92).

Variable	Health Literacy (rs)	Diabetes Self-Care Management (rs)
Health Literacy	N/A	0.64 ** [0.50, 0.75]
Income Level	0.41 ** [0.22, 0.57]	0.40 ** [0.21, 0.56]
Age	−0.36 ** [−0.53, −0.17]	−0.49 ** [−0.63, −0.31]
Gender	−0.02 (*p* = 0.873) [−0.22, 0.19]	0.11 (*p* = 0.296) [−0.10, 0.31]
Education Level	0.26 * [0.06, 0.44]	0.29 ** [0.09, 0.47]

Note: rs = Spearman rank-order correlation coefficient; values in brackets are 95% confidence intervals derived via Fisher’s r-to-z transformation. Non-significant results: gender–health literacy *p* = 0.873; gender–self-care *p* = 0.296. * *p* ≤ 0.05; ** *p* ≤ 0.01.

**Table 4:** Multiple Linear Regression Predicting Diabetes Self-Care Management from Health Literacy, Age, Income, and Education (N = 92).

Predictor	B	SE	β (std.)	*p*	95% CI for B
Health literacy (HLS-Q12)	0.088	0.023	0.355	<0.001	[0.042, 0.133]
Age	−0.035	0.009	−0.311	<0.001	[−0.053, −0.016]
Monthly income	0.00005	0.000026	0.170	0.063	[−0.000003, 0.00010]
Education level	0.214	0.124	0.144	0.088	[−0.033, 0.462]

Note: B = unstandardized coefficient; SE = standard error; β = standardized coefficient. Model R^2^ = 0.450, adjusted R^2^ = 0.424, F(4, 87) = 17.77, *p* < 0.001.

## Data Availability

The original contributions presented in this study are included in the article. Further inquiries can be directed to the corresponding author.
